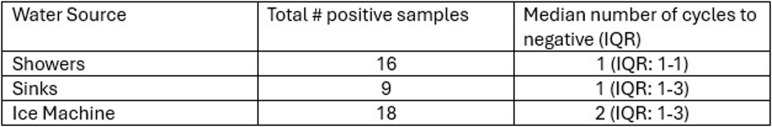# 249 Lessons Learned from Measles Exposure Response

**DOI:** 10.1017/ash.2026.10619

**Published:** 2026-06-23

**Authors:** Allison Wu, Annabella Blackman, Judy Yan, John Letson, Mini Kamboj, Tania Bubb, Shauna Usiak

**Affiliations:** 1 Memorial Sloan Kettering Cancer Center; 2 Memorial Sloan Kettering

## Abstract

**Background:** Building water systems are known reservoirs for Legionella species, which can lead to healthcare-acquired infections, particularly among immunocompromised patients. Ice machines present complex internal surfaces and components that can support biofilm formation, which makes remediation challenging. This study evaluated the number of mitigation cycles required to eliminate Legionella from hospital water sources, with a focus on ice machines. **Methods:** A retrospective review of environmental Legionella testing from 2024-2025 was conducted. This included positive results identified from ice machines, sinks, and showers through routine surveillance and outbreak investigations. For each contaminated source, the number of mitigation cycles required for each source to achieve negative results was assessed. A total of 18 ice machines and 25 sinks and showers that tested positive for Legionella species were included in the analysis. **Results:** A total of 367 water sources were sampled during the study period, including 55 ice machines and 312 sinks and showers. 25 sinks and showers tested positive (8% of total samples) over a two-year period and required a mean of 1.66 mitigation cycles, with no fixture requiring more than five cycles to achieve negative results. In comparison, 18 positive ice machines (33% of total samples) required a mean of 2.67 mitigation cycles to achieve negative Legionella results, with one machine requiring eight cycles. Overall, the ice machines required, on average, one additional mitigation cycle to achieve negative results compared to the sinks and showers. ANOVA testing revealed no statistically significant differences in the number of cycles to clear Legionella between the different sources (F(2,40)=2.65, p=0.083). **Conclusion:** Ice machines demonstrated a higher frequency of persistent Legionella positivity and required more remediation cycles compared with sinks and showers. While the results were not statistically significant, additional data may demonstrate clearer differences in Legionella clearance across different sources. The persistent positive ice machines prompted implementation of an enhanced sanitization process beyond manufacturer recommendations. This highlights the challenges associated with biofilm control in these devices. Standard cleaning practices based on ice machine manufacturer’s instructions for use (MIFU) may be insufficient at eliminating contamination. These findings support the need for enhanced mitigation strategies and routine environmental surveillance.

**Figure:**